# Probabilistic Information Modulates the Timed Response Inhibition Deficit in Aging Mice

**DOI:** 10.3389/fnbeh.2019.00196

**Published:** 2019-09-03

**Authors:** Ezgi Gür, Yalçın Akın Duyan, Fuat Balcı

**Affiliations:** ^1^Timing and Decision Making Laboratory, Koç University, Istanbul, Turkey; ^2^Koç University Research Center for Translational Medicine, Istanbul, Turkey

**Keywords:** cognitive aging, interval timing, probabilistic reasoning, temporal discrimination, temporal processing, response inhibition

## Abstract

How interval timing is affected by aging constitutes one of the contemporary research questions. There is however a limited number of studies that investigate this research question in animal models of aging. The current study investigated how temporal decision-making is affected by aging. Initially, we trained young (2–3 month-old) and old C57BL/6J male mice (18–19 month-old) independently with short (3 s) and long (9 s) intervals by signaling, in each trial, the hopper associated with the interval that is in effect in that trial. The probability of short and long trials was manipulated (0.25 or 0.75) for different animals in each age group. During testing, both hoppers were illuminated, and thus active trial type was not differentiated. We expected mice to spontaneously combine the independently acquired time interval-location-probability information to adaptively guide their timing behavior in test trials. This adaptive ability and the resultant timing behavior were analyzed and compared between the age groups. Both young and old mice indeed adjusted their timing behavior in an abrupt fashion based on the independently acquired temporal-spatial-probabilistic information. The core timing ability of old mice was also intact. However, old mice had difficulty in terminating an ongoing timed response when the probability for the short trial was higher and this difference disappeared in the group that was exposed to a lower probability of short trials. These results suggest an inhibition problem in old mice as reflected through the threshold modulation process in timed decisions, which is cognitively penetrable to the probabilistic information.

## Introduction

How temporal information processing is altered in aging is a fundamental research question (Xu and Church, 2017). The results gathered from human and animal studies targeting this very question have vastly led to equivocal conclusions regarding the health of temporal information processing in aging (e.g., Balci et al., 2009d; Turgeon et al., 2016). Most of these studies have assessed timing performance with no-to-minimal focus on how other relevant parameters such as probabilities and spatial locations are integrated into temporal information processing. In order to fill this empirical gap, the current study investigated how the core features of interval timing performance, the integration of probabilistic information into timing behavior, and the spontaneous behavioral adjustments based on previously acquired information regarding these parameters are altered in the mouse model of aging.

Anecdotal evidence based on the personal reports suggests that subjective time flows faster with aging (Friedman, 2013). Theoretic treatment of this observation coupled with convergent evidence suggesting slower information processing in old age (Salthouse, 1996; but see Starns and Ratcliff, 2010) suggests that the internal clock slows down with aging. This conclusion is supported by slower and more variable tapping in very simple tasks (i.e., unpaced finger-tapping—Vanneste et al., 2001; Turgeon and Wing, 2012). Other studies using dual-task paradigms have also demonstrated disrupted timing performance, which is attributed to the disrupted allocation of attentional resources between temporal and non-temporal aspects of the tasks (for review, see Balci et al., 2009d). These earlier studies do not address the ability of older organisms to integrate other task-relevant parameters with temporal information for guiding adaptive actions.

Our earlier work has shown that humans and mice can integrate probability information into their temporal decisions in an adaptive fashion (e.g., maximizing reward-rate; Balci et al., 2009b; Kheifets and Gallistel, 2012; Çoskun et al., 2015; Akdoğan and Balcı, 2016) and they can do this abruptly and spontaneously (Tosun et al., 2016; for a review, see Gür et al., 2018). Regarding these functional endpoints, earlier studies show that sensitivity to probabilistic information is higher in young compared to elderly (Howard et al., 2008), which becomes more pronounced with extended practice (Simon et al., 2010). Despite these differences in the utilization of probabilistic information, the ability to detect probabilities were reported to be intact in elderly when young and old participants were equated on memory encoding capacity (Spaniol and Bayen, 2005).

The current study investigated how the nature and degree of integration of probabilistic information into temporal decisions as well as the resultant timing behavior change with aging. To this end, we first trained young and old mice independently on two temporal options with different probabilities in an autoshaping setting. In order to probe and assess the timing performance of mice and their ability to modulate their timing behavior based on previously and independently experienced probabilistic and spatiotemporal relations, we introduced an experimental setting that required the spontaneous integration of these variables for adaptive anticipatory responding.

## Materials and Methods

### Subjects

Subjects were 34 experimentally naive male C57BL/6J mice purchased from Koç University Animal Research Facility. Sixteen of the mice were approximately 2 months old and 18 mice were 18 months old at the start of the experiment. Two old mice died of natural causes and could not complete the experiment. Another old mouse was excluded from the data analysis due to the lack of data points required to make the model fits. Consequently, data from 31 subjects were analyzed. Animals were housed in groups of 3–5 in polycarbonate cages (Allentown type I long individually ventilated cages) in rooms that were illuminated on a 12:12-h light:dark cycle (lights on at 6:00 AM). Subjects were tested during the light cycle on consecutive days. Three days prior to the start of the experiment, mice were subjected to a food deprivation protocol with *ad libitum* access to water. After each experimental session, they were given additional food pellets to maintain them at 85% of their free-feeding weight. All procedures reported here were approved by Koç University Animal Research Local Ethics Committee (Protocol Numbers: 2013-2 and 2014-13).

### Apparatus

Mice were tested in operant chambers with metal end walls, and transparent plexiglass side walls and ceiling. In one of the end walls, there were three illuminable food hoppers. Hoppers at the extreme sides were active to deliver 0.01 mL of diluted liquid feed (Nestlé Nutrition Isosource, vanilla flavor) as the reward and to signal time intervals. On the opposite wall, levers were retracted at all times. The nose poke hole in between the levers was used for mice to initiate the trials. Head entries to the food magazines and the nose poke hole were detected *via* IR beam break detectors. MED-PC IV software was used to run the experiment and record the data. All events were logged and time-stamped with a resolution of 10 ms. The boxes were ventilated throughout all sessions.

### Procedure

#### Training Phase

**Figure 1:**
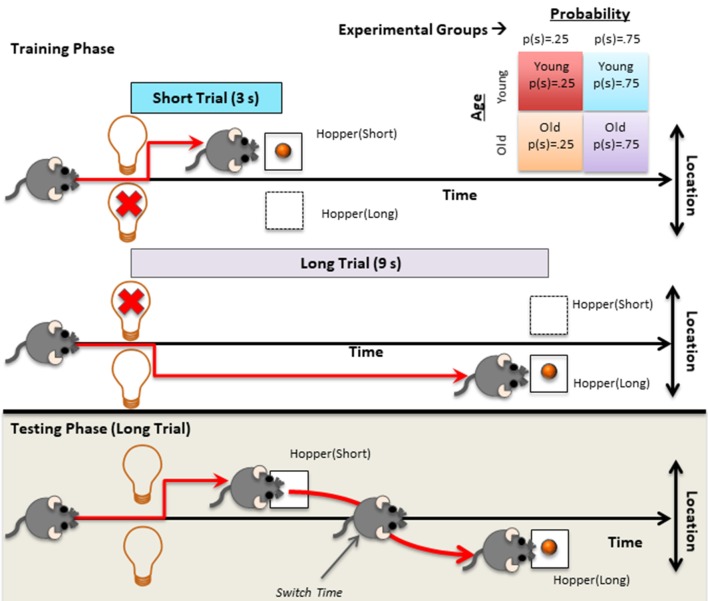
Graphical depiction of the procedures applied during training and testing along with the depiction of the typical behavior observed in the corresponding trials. Top right table depicts the four different experimental groups (2 age groups × 2 probability conditions).

Mice were trained on two types of trials, 3 s (short) trials and 9 s (long) trials, which were presented in a randomly intermixed fashion. Each feeding hopper was associated with one of the two trial types (counterbalanced between subjects). The nose poke hole was illuminated to signal that a trial could be initiated upon responding there. When the subject initiated the trial, the feeding hopper associated with the current trial type was illuminated. The light stayed on for the duration of the trial type and the reward was delivered irrespective of the subject’s response at the light offset for 6 s (autoshaping). The inter-trial interval (ITI) was fixed 30 s delay plus an exponentially distributed random variable with a mean of 60 s. Each session lasted for an hour. Regardless of the subjects’ performance to collect rewards, each subject took the first test session after 20 training sessions. Importantly, mice in each age group were divided into two groups and trained separately with the following probability conditions for trial type: *p*(short) = 0.25 and *p*(short) = 0.75. Upper panel of Figure 1 depicts the training protocol and typical behavioral pattern observed during short and long trials.

#### Test Phase

The test phase was identical to the training phase, except that instead of a single (active) hopper illumination, both feeding hoppers were illuminated for the entire duration of the trial type. Same short trial probabilities used in the training were used during testing. The test session was 2-h long. The lower panel of Figure 1 shows the long test trials and typical switch behavior observed during these trials. After the test session, subjects went through the training protocol for one daily session and testing protocol for one daily session, each lasted for 2 h, on two consecutive days.

### Data Analysis

**Figure 2:**
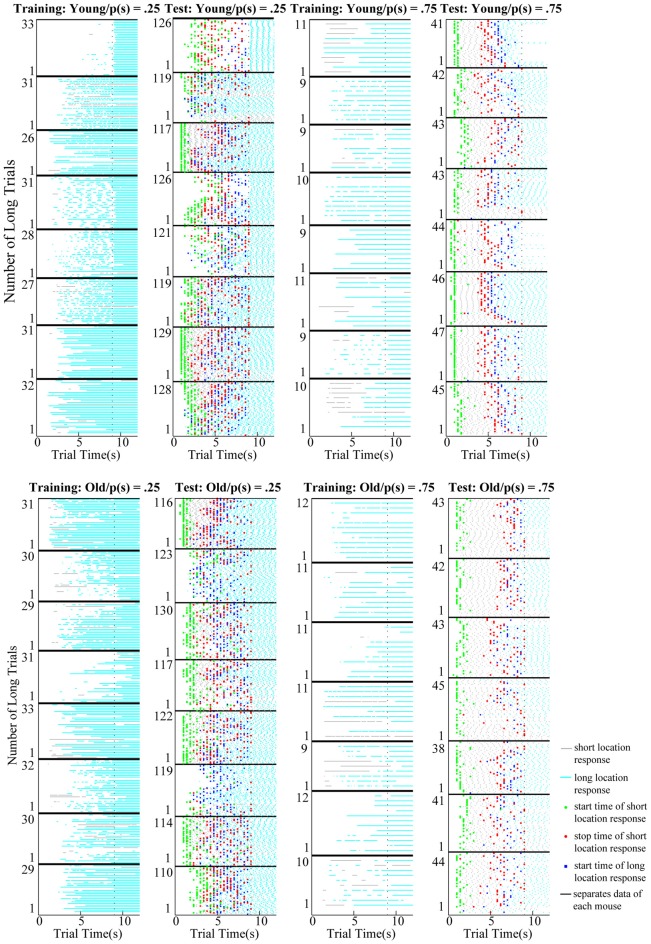
Raster plots of short (gray) and long (cyan) location responses in the long trials of training (columns 1 and 3) and test (columns 2 and 4) sessions. Upper and lower panels show the data collected from young and old mice, respectively. Horizontal tick black lines separate the data collected from each mouse. Vertical dotted black lines show the time of reward delivery in the long trials (9 s). Mice rarely switched from the short location to the long location during the long trials of the training session; however, timed switches were apparent from the outset during the long trials of testing in both age groups. Green, red, and blue dots correspond to the start time of short location responses, stop time of short location response, and start time of long location responses estimated from the single-trial analysis, respectively.

Switch latencies (or switch times) were defined as the time mice left the short duration hopper for the long duration hopper in the long trials since the illumination of the hopper(s). These latencies were used to calculate the mean switch time and coefficient of variation (CV = Standard Deviation/Mean) for each subject based on the Weibull function that fits with the data collected during two test sessions. Model fits were done using maximum likelihood estimation method. Note that in the calculation of switch rates we limited our definition of switches in the long trials to the ones which were earlier than 9 s given that switches after that point were “contaminated” by the presentation of reward in the hopper associated with the long duration. This resulted in only a few exclusions in the training phase as switch behavior was rare during this phase (two young and three old mice had relatively higher switch rates—see Figure 2). We also computed switch rates (the number of switches divided by the number of long trials) for the last training session and two test sessions combined. Two test sessions were evaluated by combining the data since the results were similar when we applied the analysis for individual test sessions. The same analysis (on switch rate and time) was conducted also for the first hour of the initial test session to be able to compare the results to those of Tosun et al.’s (2016) study. The results of this additional analysis that replicated our earlier findings are presented as Supplementary Material (SM) along with the comparable analysis of the data from Tosun et al. (2016).

Acquisition of the task during training in relation to the timing of first responses in both locations was examined in order to see whether there was a difference between the age and probability groups throughout the training. First, the time of first visits to the short location in the short trials and the time of first visits to the long location in the long trials were extracted, which were then averaged for each session and mouse. Then, the time of first visits was regressed on the session order for each mouse separately for the first short and first long location responses in the short and long trials, respectively. The acquired slopes were compared between the age and probability groups as well as between the short and long trials. Additionally, to see if there is any learning/adjustment in switch times throughout the first test session, we first regressed the switch times on their order of occurrence and compared them between the experimental groups.

We were also interested in the beginning and termination of reward anticipation in short location and the beginning of reward anticipation in the long location in the long trials of the test sessions. Since long trials terminated with reward delivery, we could not compute the termination of reward anticipation for the long location. The anticipation of reward is captured by the high state of responding at the location of reward delivery in a given trial. Time points that mark the beginning and end of the high state are referred to as start (s1) and stop (s2) times. Here, we used cumulative sum test (CUSUM) with absolute residuals to detect the trial times at which subjects transitioned from low-to-high (s1) or high-to-low (s2) state of responding at the short and long locations in each long trial (Church et al., 1994). Times that maximized the difference between high and low rates were calculated by t1(r − r1) + t2(r2 − r) + t3(r − r3) separately for the short and long location responses, respectively; t1 is the time from the beginning of trial until s1, t2 is the time between s1 and s2, t3 is the time between s2 and the end of trial, *r* is the overall mean response rate, r1, r2, and r3 are the mean response rates during t1, t2, and t3, respectively (Church et al., 1994). During the detection of start/stop times for a given trial, the algorithm detected the start and stop times of short location responses first. The search for the start time of long location responses was started from the stop time of short location responses. If there was no short response in that trial, the start time of long location responses was searched from the beginning of the trial. Any start or stop time later than 9 s (time of reward delivery) were excluded from the analysis (8% of all trials).

After the detection of start/stop times of short location responses and start times of long location responses, CV of each measure was also calculated for individual subjects. The point of maximum expectancy for the reward in the short location (i.e., middle time) was defined as the mean of start and stop times of short location responses. Difference between start and stop times of short location responses (spread) was also calculated as an index of timing uncertainty for a given trial.

The analysis of variance was used to make comparisons between age and probability groups for the slope of the first response times for training, slopes of switch times during the test, switch rates during training/testing, switch times, CV of switch times, start and stop times of short location responses. In the case of assumption violation, we used *t*-test by splitting subjects by one of the variables. We refer the readers to the SM for the statistical comparisons of age and probability conditions for CVs of start and stop times of short location responses, middle times and spread of the short location responses, start time of the long location responses and its CV. Raw data were processed using Matlab to acquire parameters for each individual subject. Statistical comparisons were conducted using SPSS 24 and JASP (JASP Team, 2019).

## Results

### Acquisition During Training and Test Phase

Comparison of the individual slopes acquired from the timing of first responses at short and long locations on the short and long trials, respectively, revealed that the (absolute value of) slopes of the first response time of short location responses was significantly higher than the slopes of the first response time of long location responses, [*F*_(1,26)_ = 14.78, *p* ≤ 0.001; *M*_short_ = −0.67 (0.06) vs. *M*_long_ = −0.41 (0.06)]. Neither the main effect of age (*F*_(1,26)_ = 1.08, *p* = 0.31) nor the probability (*F*_(1,26)_ = 3.15, *p* = 0.09) were significant. The interaction effects of trial type*age (*F*_(1,26)_ = 0.94, *p* = 0.34), trial type*probability manipulation (*F*_(1,26)_ = 2.70, *p* = 0.11), age*probability manipulation (*F*_(1,26)_ = 0.05, *p* = 0.83), and trial type*age*probability manipulation (*F*_(1,26)_ = 3.20, *p* = 0.09) on these slopes throughout the training phase were also not statistically significant.

The analysis of slopes of switch times as a function of their order of occurrence within the first test session based on conventional tests revealed that there was a significant slope in only 4 out of 31 cases. Consistently, the corresponding Bayesian analysis supported the alternative hypothesis only in six cases, and only two of these six cases were based on strong evidence while the rest had only anecdotal evidence. Comparison of these values between age and probability groups revealed that there was no main effect of age (*F*_(1,27)_ = 0.04, *p* = 0.85), main effect of probability (*F*_(1,27)_ = 1.19, *p* = 0.29), or age*probability interaction (*F*_(1,27)_ = 2.73, *p* = 0.11). Briefly, our results did not point at any age-dependent differences in the acquisition of timed responses.

### Switch Rates in Training vs. Test Sessions

Difference between the switch rates during the long trials of training and test sessions reflects whether mice can utilize information acquired during the training phase to adaptively respond in the ambiguous situation created in the test session. Raster plots show the response patterns observed in every trial both for training and testing sessions of each subject (Figure 2). During training, switch behavior was rare when only the location of reward delivery was signaled (first and third columns of Figure 2). However, in the test sessions in which both options were signaled, in the long trials mice often switched to the long location after first visiting the short location (second and fourth columns of Figure 2). We compared the difference between switch rates of training and testing as well as the age differences in the switch rates.

**Figure 3:**
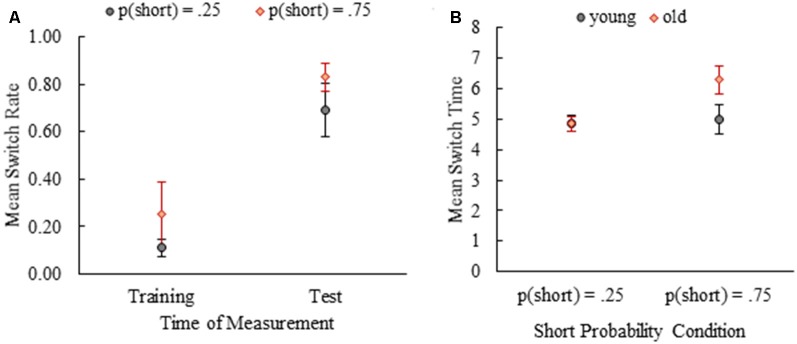
Mean switch rates for the last training session and two test sessions combined **(A)**. Mean switch times of young and old mice by short probability conditions **(B)**. Error bars show 95% confidence intervals (Mean ± 1.96*SE).

A mixed design analysis of variance (ANOVA) with age as the between-subjects factor and time of measurement (last training session vs. test sessions) as the within-subjects factor revealed a significant main effect of phase on switch rates both for *p*(short) = 0.25 [*F*_(1,14)_ = 93.66, *p* < 0.001, partial *η*^2^ = 0.87; *M*_training_ = 0.11 (0.02) vs. M_test_ = 0.69 (0.06)] and *p*(short) = 0.75 [*F*_(1,13)_ = 82.33, *p* < 0.001, partial *η*^2^ = 0.86; M_training_ = 0.25 (0.07) vs. M_test_ = 0.83 (0.03)] conditions. In other words, mean switch rates increased substantially from the training phase to the test phase in both probability conditions (Figure 3A). The main effect of age on switch rates was not significant in *p*(short) = 0.25 [*F*_(1,14)_ = 0.23, *p* = 0.64; M_young_ = 0.39 (0.05) vs. M_old_ = 0.42 (0.05)] or *p*(short) = 0.75 [*F*_(1,13)_ = 0.62, *p* = 0.44; M_young_ = 0.57 (0.06) vs. M_old_ = 0.50 (0.07)] groups. Interaction of age and time of measurement was not significant [*p*(short) = 0.25: *F*_(1,14)_ = 0.15, *p* = 0.71 and *p*(short) = 0.75: *F*_(1,13)_ = 0.87, *p* = 0.37], either. These results suggested that timed-switching was an emergent behavior due to new (ambiguous) task demands both in young and old mice regardless of probability condition [Note: probability was not included in the analysis as a factor but when it was included, nonparametric tests (Mann-Whitney *U* Test) were run due to the violation of homogeneity of variance assumption. The effect of probability on switch rate was not significant (*p*_*training*_ = 0.47, *p*_test_ = 0.09)]. The same results held even when the data from the first hour of the initial test session was analyzed between the phases and age groups (for details see Supplementary Material).

### Switch Times in Test Sessions

We also analyzed the effect of age and probability manipulation on switch time as well as their interaction given that timed behavior might have deteriorated with age and previous work showed that probability manipulation exerts an effect on switch times. A 2 × 2 ANOVA with age (young vs. old) and probability condition (0.25 vs. 0.75) as between-subject factors revealed main effects of age (*F*_(1,27)_ = 12.09, *p* = 0.002, partial *η*^2^ = 0.31) and probability manipulation (*F*_(1,27)_ = 18.31, *p* < 0.001, partial *η*^2^ = 0.40). Old mice (*M* = 5.52, SE = 0.23) had later switch times compared to young mice (*M* = 4.93, SE = 0.13). Switch times were later for the mice in *p*(short) = 0.75 condition (*M* = 5.60, SE = 0.23) compared to *p*(short) = 0.25 condition (*M* = 4.85, SE = 0.09). Importantly, these main effects were coupled with a significant interaction of age and probability, *F*_(1,27)_ = 12.52, *p* = 0.001, partial *η*^2^ = 0.32. In *p*(short) = 0.25 condition, mean switch times of young and old mice did not differ, MD = 0.01, SE = 0.26, *p* = 0.97. In *p*(short) = 0.75 condition, on the other hand, the mean switch time of old mice was significantly later than mean switch time of young mice, MD = −1.29, SE = 0.27, *p* < 0.001. Figure 3B shows the mean switch times for both probability conditions by age. These results revealed an age difference in timed switching behavior moderated by training with different trial probabilities. When we analyzed the switch times from the first hour of the initial test session, the effect of probability manipulation was evident, but we did not observe an age and probability interaction (see Supplementary Material).

As an indicator of temporal precision, we analyzed the CV values. A two-way ANOVA was conducted to see the effects of age and probability on CV of the switch times. The results showed that there was a significant main effect of probability condition; mice in *p*(short) = 0.25 condition (*M* = 0.39, SE = 0.02) had higher CV on average compared to *p*(short) = 0.75 condition (*M* = 0.20, SE = 0.01) regardless of the age (*F*_(1,27)_ = 63.02, *p* < 0.001, partial *η*^2^ = 0.70). There was no main effect of age, *F*_(1,27)_ = 0.08, *p* = 0.79 or interaction effect between age and probability manipulation, *F*_(1,27)_ = 0.12, *p* = 0.74. These results suggested that the temporal precision of young and old mice was comparable.

### Timed Anticipatory Responses During Test Session

**Figure 4:**
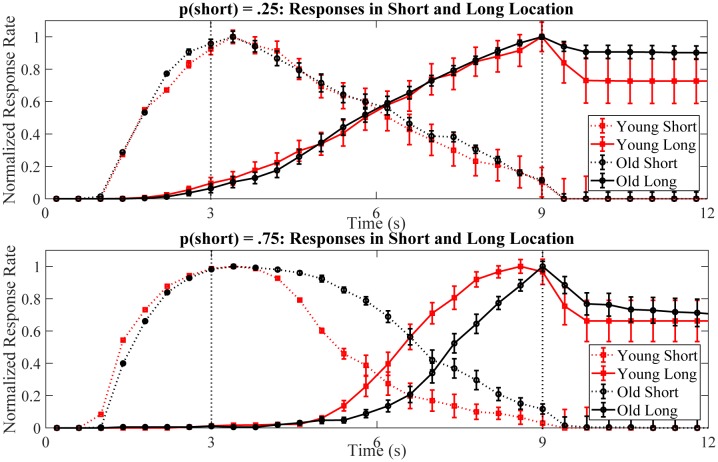
Normalized averaged response curves for short and long location responses during long trials of test sessions for young (red) and old (black) mice in different probability conditions. Dotted vertical lines represent the time of reward delivery in short and long locations; however, note that there was no reward delivery in the short location during long trials. Early in the trial, the normalized response rate was higher for the short location (dotted black and red curves) and it peaked around 3 s. For the long location, the normalized response rate increased (solid black and red curves) later in the trial. Error bars show the standard error of the actual mean values.

Not only the timed-switching pattern of a subject in the timed switch task but also the timing of short and long location responses separately might allow us to characterize anticipatory behavior in more detail to investigate age differences in interval timing. To do so, we compared several measures of timing behavior that reflect distinct processes within interval timing between age and probability groups. The analysis was limited to long trials during testing, allowing us to characterize timing behavior during reward omissions for short location responses, and prior to reward presentation for long location responses. Figure 2 provides each subject’s data on a trial-by-trial basis showing short location responses and long location responses during training and test sessions as well as start and stop times extracted for the test phase. Figure 4 presents the normalized averaged response curves for the short and long location responses in order to provide an overall idea about the timing performance of young and old mice.

**Figure 5:**
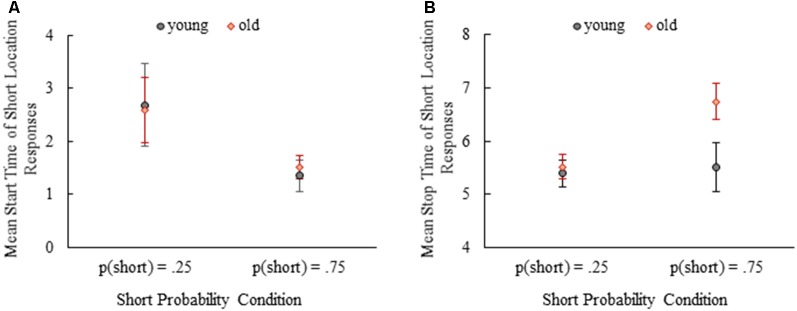
Mean start time of short location responses **(A)** and mean stop time of short location responses **(B)** depending on age and probability conditions. Error bars show 95% confidence intervals (Mean ± 1.96*SE).

Comparisons of start times of the short location responses were done between young and old mice and probability conditions (Figure 5A) by splitting data for one of the variables due to the violation of homogeneity of variance assumption. When the data were split by age, we found that start times of the short location responses were later in *p*(short) = 0.25 condition compared to *p*(short) = 0.75 condition both in young (*t*_(8.90)_ = 3.15, *p* = 0.01) and old mice (*t*_(8.81)_ = 3.22, *p* = 0.01) on average. After splitting the data by probability condition, there were no differences between young and old mice in *p*(short) = 0.25 (*t*_(14)_ = 0.20, *p* = 0.85) and *p*(short) = 0.75 (*t*_(13)_ = −0.89, *p* = 0.39) conditions. Mean start time of the short location responses were 2.68 (SE = 0.40) and 1.35 (SE = 0.15) for young mice and 2.58 (SE = 0.31) and 1.52 (SE = 0.11) for old mice in *p*(short) = 0.25 and *p*(short) = 0.75 conditions, respectively.

The effect of age and probability was also examined for the stop times of short location responses (Figure 5B). Mean stop time of short location responses was significantly earlier for young mice compared to old mice, *F*_(1,27)_ = 16.52, *p* < 0.001, partial *η*^2^ = 0.38. There was also a significant main effect of probability manipulation showing that mean stop time of short location responses were earlier in *p*(short) = 0.25 condition compared to *p*(short) = 0.75 condition, *F*_(1,27)_ = 16.28, *p* < 0.001, partial *η*^2^ = 0.38. Importantly, we found a significant interaction of age and probability on the mean stop time of short location responses, *F*_(1,27)_ = 11.11, *p* = 0.003, partial *η*^2^ = 0.29. Simple effect analysis showed that the mean stop time of short location responses were comparable between young and old mice in *p*(short) = 0.25 condition, MD = −0.12, SE = 0.23, *p* = 0.60. However, aged mice stopped responding in the short location significantly later than the young mice in *p*(short) = 0.75 condition, MD = −1.24, SE = 0.24, *p* < 0.001. These findings can also be observed at normalized averaged response curves in Figure 4 (compare two panels).

## Discussion

This study investigated age differences in temporal decision-making, anticipatory timing behavior, and spontaneous integration of task parameters into temporally controlled goal-directed responses. Our results revealed that both young and old mice were able to adopt a novel action plan during testing (i.e., timed switching) that required them to take into account the previously and independently learned temporal characteristics and probabilities of reinforcement at two different choice locations. This observation was evident in significantly higher timed switching rate found in test sessions compared to training regardless of the age group (irrespective of the duration of testing). Age differences were observed in switch times and stop times for short location timed responses that were moderated by the probabilities of options. Specifically, when the probability of reward delivery after the short interval was high; old mice persevered more at the short-latency option. As expected, this difference between the age groups was reflected in both switch time and stop times for the short location responses; two highly correlated measures (*r* = 0.95, *N* = 31, *p* < 0.001).

Observed response perseveration shows parallelism to other findings reported in the literature in relation to neurodegenerative conditions (e.g., Huntington’s Disease, Balci et al., 2009a), disruptive effect of scopolamine (typically used as a model of dementia; Ebert and Kirch, 1998) on the peak interval responding (Abner et al., 2001; Balci et al., 2008) and it is consistent with the effect of aging on single peak procedure (see Figure 4 bottom panel in Church et al., 2014). On the other hand, interestingly the observed disruption of timing behavior in terms of the termination of timed responses was present only when the probability was an independent source of bias favoring the reward delivery at the short-latency option. Combined with our previous findings outlined above, this empirical observation suggests that the inhibition deficit can be rescued when the likelihood of the corresponding option does not favor the option associated with the to-be inhibited action (i.e., *p*(short) = 0.25) possibly as an independent source of cognitive control.

In line with this rationale, previous studies on behavioral inhibition have also suggested that the manipulation of stimulus probabilities that signal to act or not to act alters the behavioral inhibition performance by modulating the strength of response preparation process (e.g., Bruin and Wijers, 2002). Therefore, we suggest that the inhibitory control integrates multiple sources of information including different quantities/dimensions such as time and probability.

Our empirical observations are also consistent with the weakened inhibitory control theory of cognitive aging (Kramer et al., 1994; Potter and Grealy, 2008; Coxon et al., 2012). For instance, older individuals have also been shown to persevere with a previous rule on the Wisconsin Card Sorting task (Ashendorf and McCaffrey, 2008). Possibly reflecting similar perseverative tendencies, older rats and mice were found to exhibit lower spontaneous alternation performance compared to young rats (Willig et al., 1987) and mice (Stone et al., 1992). These results point to cognitive perseveration as the possible common factor. Inhibitory (action) control in aging is typically evaluated in relation to the signaled termination of an initiated response (e.g., stop signal reaction time task). These studies reported a slower reaction to stop signals for the elderly (Bruin and Wijers, 2002; van de Laar et al., 2011). A recent meta-analytic study also concluded that inhibition deficits due to aging are specifically seen in the form of suppressing dominant response rather than ignoring distractor information or response interference (Rey-Mermet and Gade, 2018). In our case, such inhibition problem in old mice was evident in the delayed switch and stop times (and thus an ongoing response) specifically when the trial probability favored the short option, which presumably turns the ongoing responses in the short location into a dominant response that could not be inhibited by older mice.

Several different neural mechanisms have been implicated for such inhibitory control deficits. However, the current study cannot differentiate if the observed deficit in inhibitory control is due to altered functional connectivity in cortico (right inferior frontal gyrus and presupplementary motor area)-subthalamic nucleus (Coxon et al., 2012), fronto-basal ganglia (Suchy et al., 2013; for instance in relation to hyperkinetic perseveration; Goldberg, 1986) or another circuit that has been implicated with inhibitory control deficit in aging. Future studies needed to elucidate the role of these candidate neural circuitries for the observed effects and their relationship to timing behavior.

Our results also show that the processes of spontaneous integration of information that were gathered independently during the prior experience were not affected by cognitive aging. All the indices of timing performance (except for the stop times for short location) were comparable between the two age groups. There was an interaction effect of age and probability on the stop times of the short location responses. A disruption in the core timing component (i.e., clock) or memory would predict shifts in both the start and stop times by the same amount and in the same direction, which was not the case in our study.

Observed differences in our study are most likely related to the factors that relate to the decision component of interval timing. Specifically, old mice set a higher threshold (criterion) for response terminations when the probability of the short trial was higher, which in turn resulted in later response terminations and delayed switch times. Given that there was no difference between the age groups in terms of the start times, we propose that in our case, the modulation of timed responses by probabilistic information was (partially) *via* altering the threshold setting of specifically when to terminate an ongoing response. This claim is supported by the independence of decision processes that guide the initiation and termination of timed responding (Church et al., 1994; Balci et al., 2009c; MacDonald et al., 2012). Finally, we observed higher CV of the start times of the short location responses in older mice, which might be due to the higher trial-to-trial variation in the motivational states of the old mice (e.g., Balcı, 2014); however, this possibility requires further investigation.

The current study also constituted the direct replication of our previous work (Tosun et al., 2016—see Supplementary Material for the results of the comparable statistical analysis) when the first hour of the initial testing was analyzed to match the 1-h long testing in the previous work. Consistent with Tosun et al. (2016), we found that during the test sessions mice can immediately and rapidly integrate previously learned task parameters to plan and guide their choice behavior. The resultant timing behavior was sensitive to probabilistic manipulations and did not change over the course of testing for the majority of the cases (both for the entire 2 h long first test session and during its first 1 h). These results challenge the theoretical approaches that solely rely on gradual learning based on trial-by-trial response-outcome experiences (for review, see Gür et al., 2018; Malet-Karas et al., 2019).

One of the limitations of the current work is that animals received feedback (in the form of reinforcement) for correct responses during testing, which introduces a possible means for animals to learn based on differential reinforcement (albeit this learning would still have to be very abrupt to account for our observations). Future research can omit reinforcement from all test trials for a cleaner characterization of the emergent behavior. The use of a single set of intervals also limits the generalizability of our conclusions to other intervals. Finally, given our experimental design, we do not know how much pre-training would be necessary to observe the abrupt emergence of timed-switching behavior. These are issues that can be addressed by future research.

## Author’s Note

This study will be a part of Ezgi Gür’s PhD thesis.

## Data Availability

The datasets generated for this study are available on request to the corresponding author.

## Ethics Statement

This study was carried out in accordance with the national ethical guidelines for the animal research. The protocol was approved by the Koç University Animal Research Local Ethics Committee (Protocol Numbers: 2013-2 and 2014-13).

## Author Contributions

FB, EG and YD conceived and designed the experiment. EG and YD performed the experiments. EG and FB analyzed the data. EG, YD and FB wrote the manuscript.

## Conflict of Interest Statement

The authors declare that the research was conducted in the absence of any commercial or financial relationships that could be construed as a potential conflict of interest.
