# Supplementary material for: Probabilistic Information Modulates the Timed Response Inhibition Deficit in Aging Mice
Source: Front Behav Neurosci. 2019 Sep 3;13:196. doi: 10.3389/fnbeh.2019.00196 (PMC6734164; doi:10.3389/fnbeh.2019.00196)
Supplement: Supplementary file 1 [file Presentation_1.pdf]

Supplementary Material

Probabilistic Information Modulates the Timed Response Inhibition Deficit in Aging Mice

Ezgi Gür[1,2], Yalçın Akın Duyan[1,2], Fuat Balcı[1,2*]

[1]Timing and Decision Making Laboratory, Koç University, Istanbul, Turkey

[2] Koç University Research Center for Translational Medicine, Istanbul, Turkey

*** Correspondence:**
Dr. Fuat Balcı
fbalci@ku.edu.tr

Supplementary Material

1 Switch Rate and Switch Latencies (Times) in the First Hour of the Initial Test Session

Due to the insufficient number of observations, 3 mice from the young group and 4 mice from the old group were not included in the analysis of the first hour of the initial test session. With the remaining subjects, there were 13 mice in the young group, 11 mice in the old group. First, we conducted a mixed ANOVA to compare switch rates between phases (last the training session vs. the first hour of the first test session) and age (young vs. old) groups. Results showed that mice switch from the short location to the long location significantly more during the first hour of the first test session ($M = .72$, $SE = .04$) compared to last training session ($M = .22$, $SE = .05$), $F(1, 22) = 104.34$, $p < .001$, partial $\eta^2 = .83$. Main effect of age ($F(1,22) = .00$, $p = 1.00$) and age by phase interaction ($F(1,22) = .41$, $p = .53$) were not significant.

Comparison of switch times between age and probability groups revealed that there was a significant main effect of probability, $F(1,20) = 12.96$, $p = .002$, partial $\eta^2 = .39$. As expected, switch times were later in p(short) = .75 condition ($M = 5.42$, $SE = .17$) compared to p(short) = .25 condition ($M =4.41$, $SE = .23$). Main effect of age ($F(1,20) = 2.43$, $p = .14$) and age by phase interaction ($F(1,20) = .22$, $p = .65$) were not significant.

Adjustment in switch times during the first hour of the test session was also examined by regressing the switch times on their order of occurrence for each mouse. Results revealed that there was no evidence for adjustment in majority of the cases. Slopes were significant in 8 out of 24 cases, and only in 4 of these cases we found moderate to strong evidence for a slope different from 0.

2 Results of Tosun et al. (2016) for Comparison to the Results of Current Study

Two experiments were run for this study. There were 15 mice in the first experiment and 12 in the second experiment. In the first experiment, short trials were presented by .25, .50, and .75 probability for three different groups. In the second experiment, presentation probability of short trial was determined as .50 and .75 for two groups. The analysis of the data from Tosun et al. (2016) based on parametric tests (as in the current study) revealed results that are comparable to the results of the current study. We provide the results of these analyses below.

Experiment 1: Comparison of switch rates between the last training and first test sessions revealed that switch rates increased substantially from training to test phase all probability conditions (p(short) = .25: $t(4) = -17.95$, $p < .001$; p(short) = .50: $t(4) = -5.91$,$p < .01$; p(short) = .75: $t(4) = -5.27$, $p < .01$), consistent with the increase observed in the current experiment. Comparison of switch times between probability conditions revealed that probability manipulation significantly affected the switch times, $F(2,12) = 14.43$, $p < .001$. Comparison of probability conditions showed that average switch time of p(short) = .75 condition was significantly later than the average switch time of (short) = .25 condition ($t(8) = -5.19$, $p < .01$). Differences observed between p(short) = .25 and p(short) = .50 ($t(8) = -2.86$, $p < .05$), and p(short) = .50 and p(short) = .75 ($t(8) = -2.68$, $p < .05$) did not hold after Holm-Bonferroni corrections.

Experiment 2: Switch rate comparison between the long trials of last training and first test sessions showed that switch rates were significantly higher during testing than training both in p(short) = .50 ($t(5) = -3.70$, $p < .05$) and p(short) = .75 ($t(5) = -7.53$, $p < .001$) conditions. Furthermore, in this experiment, there were probe trials for short and long trials without reinforcement. Provided that short probe trial duration was 9 second like the long trial durations, we also compared the switch rates from the last five training sessions to the switch rates from the long trials of the first test session (last five sessions were used to have enough trials for a meaningful comparison since probe trials constituted only 25% of presented trials for each option). Again, we found that switch rates were higher during testing than training in both probability conditions (p(short) = .50: $t(5) = -4.93$, $p < .01$; p(short) = .75: $t(5) = -7.15$, $p < .001$). This comparison showed that mice preferred to stick with the signaled short option during training phase even if there was time to explore the other option, which was not active. Switch times were comparable between p(short) = .50 and p(short) = .75 conditions, $t(10) = .07$, $p = .95$.

Note: Results of these experiments were published in Tosun, T., Gür, E., & Balcı, F. (2016). In Tosun et al. (2016), we adopted a conservative approach and analyzed the data with non-parametric tests, the results of which are comparable to the results of the current study. Here the same data from Tosun et al. (2016) were reanalyzed with parametric tests, which provided nearly identical results to those reported in the original manuscript and are also comparable to the results of the current study. These observations point to the robustness of our original findings reported in Tosun et al. (2016).

3 Analysis of Complementary Measures

CV of the start time of short location responses: We found the main effects of age ($F(1,27) = 6.94$, $p = .01$, partial $\eta^2 = .20$) and probability ($F(1,27) = 6.98$, $p = .01$, partial $\eta^2 = .20$) on the CV of the start time of short location responses. Mean CV of the start time of short location responses was lower for young mice (M = .34, SE = .02) compared to old mice (M = .43, SE = .03) independent of the probability conditions. Additionally, the CV of the start time of short location responses was lower for p(short) = .75 condition (M = .33, SE = .02) compared to p(short) = .25 condition (M = .43, SE = .03). The interaction effect of age and probability was not significant, $F(1,27) = .002$, $p = .96$.

CV of the stop time of short location responses: The effect of probability manipulation was also evident on mean CV of the stop time of short location responses, $F(1,27) = 37.91$, $p < .001$, partial $\eta^2 = .58$, with higher mean CV for mice in p(short) = .25 condition (M = .35, SE = .02) compared to p(short) = .75 condition (M = .22, SE = .02) independent of age. The effect of age ($F(1,27) = 1.08$, $p = .31$) or the interaction between age and probability ($F(1,27) = .67$, $p = .42$) were not statistically significant.

Middle time and spread of short location responses: Middle and spread values of short location responses derived from start and stop times were also compared between age and probability groups. There were no main effect of age ($F(1,27) = 3.60$, $p = .07$), probability ($F(1,27) = 1.96$, $p = .17$), or interaction of age and probability ($F(1,27) = 3.38$, $p = .08$) on the middle times of short location responses (See the peak location of normalized response curves for short location responses in Figure 4 in the main text). Comparisons of spread values were done with separate independent t-tests after splitting data by probability or age due to assumption violations for running ANOVA. When we split data by the probability, we found that the spread of the short location responses of young mice (M = 2.71, SE = .36) was not different from the spread of the short location responses of old mice in

p(short) = .25 condition [(M = 2.93, SE = .25), t(14) = -.51, p = .62]. On the other hand, young mice (M = 4.16, SE = .12) had narrower spread compared to old mice (M = 5.23, SE = .25) in p(short) = .75 condition, t(8.69) = -3.94, p = .004. When comparisons were done for probability conditions after splitting data by age, the spread of the short location responses in p(short) = .25 condition was significantly narrower than the spread of the short location responses in p(short) = .75 condition both in the young (t(8.46) = -3.83, p = .005) and old mice (t(13) = -6.47, p < .001). However, note that significant differences observed between the groups for the spread value depend on the start and stop times as it is calculated as the difference between them.

Start times of the long location responses: Comparison of the start times of the long location responses between age and probability conditions revealed a main effect of probability, F(1,27) = 19.09, p < .001, partial η^2 = .41. The mean start time of the long location responses in the p(short) = .25 condition (M = 5.80, SE = .19) was significantly earlier than the mean start time of the long location responses in the p(short) = .75 condition (M = 6.79, SE = .14). The mean start time of the long location responses were not significantly different between young and old mice, F(1,27) = .94, p = .34. The interaction of age and probability was not significant, F(1,27) = 3.01, p = .09. CV of the start time of long location responses also differed between probability conditions, F(1,27) = 29.00, p < .001, partial η^2 = .52. The CV values was higher in the p(short) = .25 condition (M = .28, SE = .02) than in the p(short) = .75 condition (M = .16, SE = .01). There was no effect of age or the interaction effect of age and probability on CV of the start times of the long location responses (age: F(1,27) = .50, p = .49, age*probability: F(1,27) = .06, p = .81).

Reference

Tosun, T., Gür, E., and Balcı, F. (2016). Mice plan decision strategies based on previously learned time intervals, locations and probabilities. Proc. Natl. Acad. Sci. U S A 113, 787–792. doi: 10.1073/pnas.1518316113
